# Advancements in nanoparticles-based therapeutic approaches for osteosarcoma: Insights from catechins-modified selenium-doped hydroxyapatite: A review

**DOI:** 10.1097/MD.0000000000041489

**Published:** 2025-02-14

**Authors:** Tao Degang, Xu Wei, Shen Jianying, Lai Aining, Jiang Chenyu, Li Haitang, Zhou Rong

**Affiliations:** a Department of Orthopedics, 72nd Group Army Hospital of the PLA, Huzhou, P.R. China; b Department of Orthopedics, The 901 Hospital of Joint Logistics Support Force, Hefei, P.R. China; c Department of Otorhinolaryngology, Huzhou Central Hospital, Huzhou, P.R. China.

**Keywords:** catechins-modified selenium-doped hydroxyapatite, nanoparticles, osteosarcoma, therapeutic approaches

## Abstract

Osteosarcoma is common in all age groups, and a multifaceted strategy that includes radiation therapy, surgical intervention and chemotherapy remains the conventional treatment for osteosarcoma. Existing therapies typically result in recurring malignancies and postsurgical bone abnormalities, necessitating novel strategies for targeted drug administration and bone defects. The most significant components that are crucial for maintain strong bones include trace elements, calcium, selenium, and vitamins K and D. A deficiency in selenium advances the risk of cancer in many organs, including the bones. The progression of an effective technique such as a “local delivery system” is required to efficiently deliver the antioxidant to the targeted tissues for treatment as the circulatory system is unable to convey an adequate concentration of catechin to the regions of bone abnormalities. In this regard the combination of selenium and catechin with mesoporous hydroxyapatite nanoparticles displays promise as a nanoscale delivery method, offering an ideal approach to use it for the treatment and prevention of bone-related diseases. Therefore, this review mainly focusing in exploring the therapeutic potential of catechins-modified selenium-doped hydroxyapatite nanomaterials, chitosan–PEG–folate–Fe (III) complexes as nanocarriers for epigallocatechin-3-gallate, and catechin-conjugated mesoporous hydroxyapatite nanoparticle, highlighting their novel functions as nano-antioxidants with improved osteogenic characteristics in osteosarcoma treatment.

## 1. Introduction

**Figure 1.**
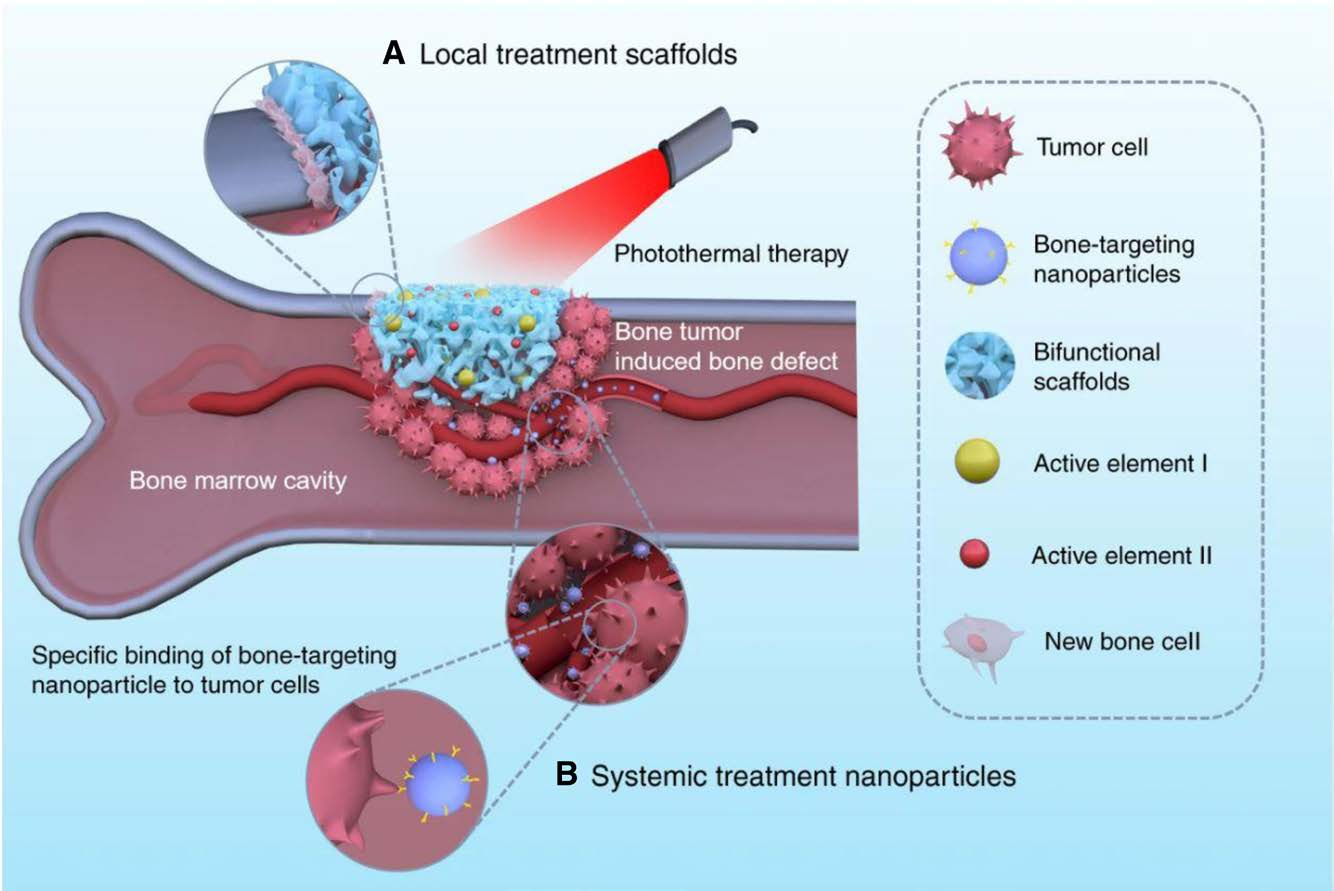
Represent systemic treatment and local treatment strategies for bone tumor. Reprinted from Ref. ^[14]^. https://doi.org/10.1038/s41413-021-00139-z, which is licensed under a Creative Commons Attribution 4.0 International License.

Osteosarcoma, which is common in children and teenagers presents a number of difficulties because of its high death rate and tendency to spread to the lungs.^[1,2]^ The conventional treatment to manage this malignancy entails a multifaceted strategy that includes radiation therapy, surgical intervention and chemotherapy.^[3,4]^ Existing therapies typically result in recurring malignancies and postsurgical bone abnormalities, necessitating novel strategies for targeted drug administration and bone defects.^[5–8]^ The most significant components that are crucial for maintain strong bones include trace elements, calcium (Ca), selenium (Se), and vitamins K and D.^[9,10]^ A deficiency in Se advances the risk of cancer in many organs, including the bones. To address skeletal inadequacies following tumor removal, it is necessary to improve the formulation of bone graft replacements with a variety of properties, including biocompatibility, osteoconductivity, sufficient mechanical strength, and biodegradability. Bioceramics, particularly hydroxyapatite (HAP), have gained significant attention and are frequently employed as bone transplant alternatives. By utilizing these strategies, the bioactive bone scaffold serves as a tailored drug delivery system, controlling the release of a variety of physiologically active substances. These substances are necessary for enhancing the mechanism of bone healing.^[11]^ Green tea catechins, particularly epigallocatechin gallate (EGCG), have anticancer effects that label multiple signaling pathways, inhibit tumor development, promote apoptosis, and make cells more susceptible to traditional therapies.^[12,13]^ The progression of an effective technique such as a “local delivery system” is required to efficiently deliver the antioxidant to the targeted tissues for treatment as the circulatory system is unable to convey an adequate concentration of catechin to the regions of bone abnormalities (Fig. 1). In this regard the combination of selenium and catechin with mesoporous hydroxyapatite (MHAP) nanoparticles displays promise as a nanoscale delivery method, offering an ideal approach to use it for the treatment and prevention of bone-related diseases (Fig. 1).^[14]^ Moreover, encapsulation of EGCG in nanocarrier systems seems to be a promising method to prevent its breakdown under physiological conditions. Therefore, this review mainly focusing in exploring the therapeutic potential of catechins-modified selenium-doped hydroxyapatite (CC/Se-HAp) nanomaterials, chitosan–PEG–folate–Fe (III) complexes as nanocarriers for epigallocatechin-3-gallate, and catechin-conjugated mesoporous hydroxyapatite nanoparticle, highlighting their novel functions as nanoantioxidants with improved osteogenic characteristics in osteosarcoma treatment.

## 2. Nanoscale CC/Se-HAp nanocomposites in osteosarcoma and osteogenesis

**Figure 2.**
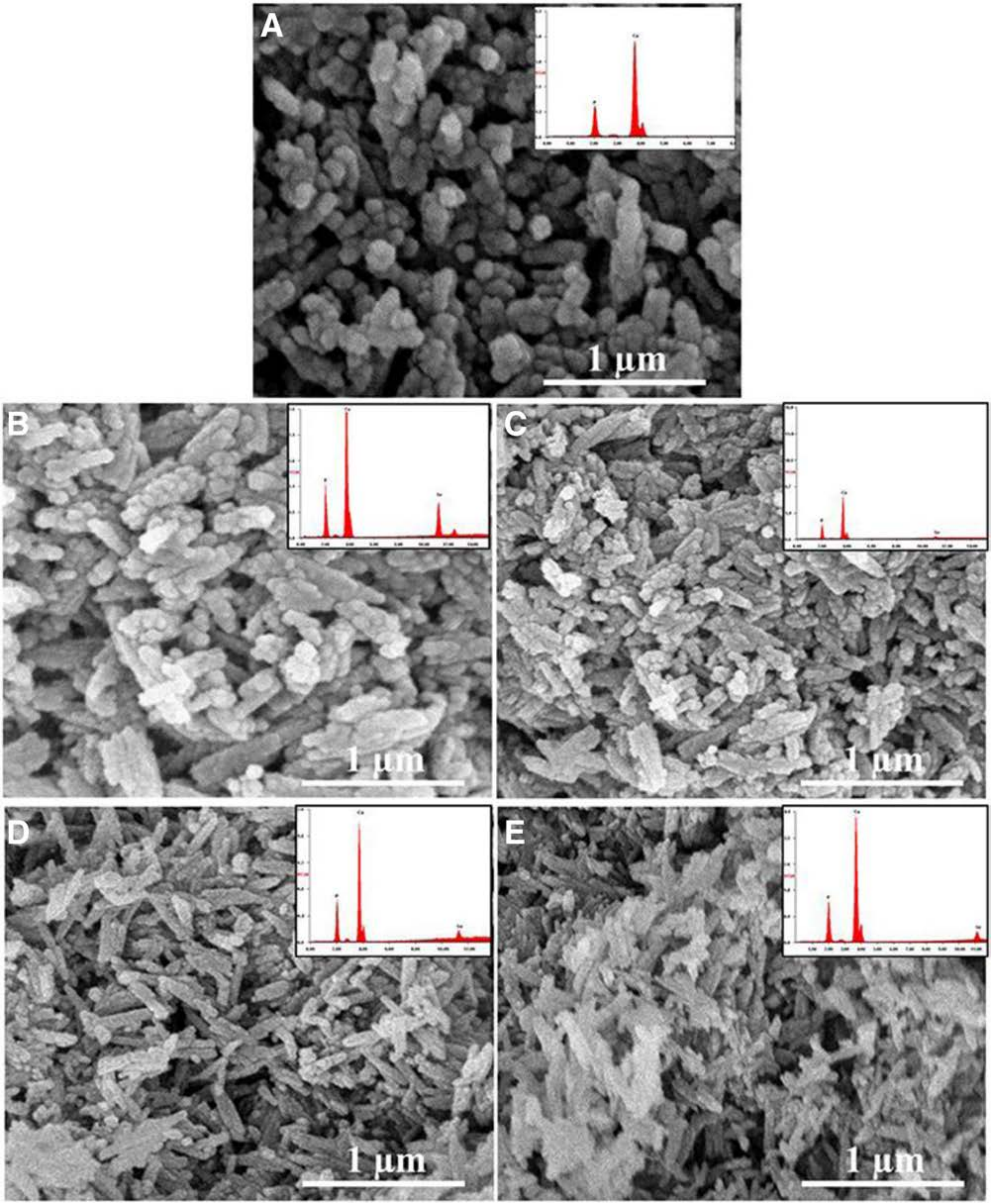
Showing the structure/shapes obtained from scanning electron micrographs and X-ray fluorescence (XRF) of catechin-modified hydroxyapatite nanocomposites designed for bone cancer treatment. Reprinted from Ref. ^[15]^. doi: 10.3389/fonc.2019.00499, which is licensed under a Creative Commons Attribution 4.0 International License.

As represented in Figure 2, CC/Se-HAp nanocomposites were prepared and studied for their physicochemical and biological characteristics, demonstrating better anticancer efficacy compared to Se-HAp nanoparticles.^[15]^ These 60 ± 15 nm-sized nanoscale HAp particles served as an excellent nanocarrier for anticancer drug delivery and an ideal template for bone formation.^[16]^ CC/Se-HAp inhibited tumor development in a P53-mediated mechanism via the production of reactive oxygen species. The study demonstrated similar cytotoxicity to nanoparticles <100 nm, highlighting their efficacy in cell endocytosis. Furthermore, the cytotoxicity of CC/Se-HAp nanomaterials was comparable to the cytotoxicity of nanoparticles with a size <100 nm exhibiting superior cell endocytosis efficiency than those with a larger size.^[17]^ The needle-like HAp nanoparticles successfully stimulated osteogenesis and triggered cell death, suggesting the involvement of Se and catechin nanoscale molecules. Notably, CC/Se-HAp preferentially stimulated the production of reactive oxygen species in N -methyl-N′-nitro-N-nitrosoguanidine/human osteosarcoma cells, suggesting that it can be used to treat osteosarcoma.^[18]^ It is interesting fact that antioxidant and osteogenesis correlate and are thus interdependent. Increased oxidation reduces the formation of bone because enhanced oxidation is directly related to cell death or destabilizing cellular machinery. Rapid internalization into N -methyl-N′-nitro-N-nitrosoguanidine/human osteosarcoma (HOS) cells is seen in TEM micrographs, which is consistent with the nanomaterial’s progressive lysosomal breakdown.^[19]^ When applied to osteosarcoma, CC/Se-HAp nanoparticles show strong targeted therapy with little adverse effects on normal stem cells. CC/Se-HAp nanoparticles had showed a strong targeted effectiveness against osteosarcoma while having a few side effects on normal stem cells, indicating their promise as a viable therapy strategy.^[20]^

## 3. Structure and composition of catechin-conjugated mesoporous hydroxyapatite nanoparticle

Catechin, a flavonoid antioxidant, was linked to mesoporous hydroxyapatite nanoparticles to create a novel type of antioxidant known as a “nanoantioxidant” with improved antioxidant and osteogenic properties. Cat@MHAP, a nanoantioxidant material, exhibits a rod-like structure and positive charge. Catechin released in malignant cells significantly improved their therapeutic index. Cat@MHAP had strong radical trapping capacity, effectively quenching hydroxyl and superoxide radicals.^[20]^ As reported by Khan et al’ aqueous precipitation method is used to prepare the pristine hydroxyapatite nanoparticles by sonication technique. Calcium nitrate tetrahydrate (Ca(NO_3_)_2_·4H_2_O) is mixed with ammonium hydrogen phosphate ((NH_4_)_2_HPO_4_), and then ammonium hydroxide (NH_4_OH) is added to prepare the solution.^[15]^ The basic polymorphic structure of HAp shows characteristic peaks, where intense and sharp peaks confirms its crystalline nature as detected X-ray diffraction (XRD). In the case of doping selenium (Se-Hap) and catechins (CC/Se-Hap) a single phase with a hexagonal structure is shown.

The XRD pattern of MHAP nanoparticles indicated a single-phase hydroxyapatite structure, as evidenced by well-aligned JCPDS values (09-0432). After catechin functionalization, the hydroxyapatite-related diffraction peaks in the Cat@MHAP XRD pattern remained unaltered, suggesting that structural integrity was preserved. Furthermore, the field-emission scanning electron microscopy picture revealed that the form and structure of MHAP nanoparticles were unaffected following catechin alteration. Energy dispersive spectroscopy confirmed the composition of Cat@MHAP nanoparticles, indicating the presence of Ca, P, O, C, Si, and N inside the structure. Following catechin conjugation on the nanoparticle surface, the positive charge magnitude dropped to +16.5 mV. The quantification of catechin loaded onto the nanoparticle surface was measured by the decline in catechin concentration in the supernatant, resulting in an estimated amount of 60 g/g nanoparticles. The nanoparticles’ surface functionalization with APTES resulted in a change to positive surface charge values (+28.24 mV), suggesting the introduction of NH_2_ groups. This reduction indicates that catechin was successfully conjugated onto MHAP nanoparticles.^[21]^ The effective integration of catechin into MHAP nanoparticles, as evidenced by structural and morphological integrity and quantitative studies, underlines Cat@MHAP’s promise adaptability for a wide range of biological applications.^[22]^

## 4. Release of effective component from nanocomposite

It is pivotal to examine the release dynamics of catechin from Cat@MHAP nanoparticles in the tumor microenvironment. The tumor microenvironment is characterized by an acidic extracellular pH (6.5–6.9) and increased proteolytic activity, generating a suitable “tumor-friendly niche,” which facilitates local invasion, tissue remodeling, and angiogenesis. Considering the conjugation of catechin by the amide bond onto the nanoparticle surface it is speculated that proteases present in the tumor cell microenvironment can catalyze the hydrolysis of the peptide link, resulting in catechin release. The investigation of catechin release dynamics involved testing a release medium with changing pH values, both with and without protease, matching the circumstances of the tumor microenvironment. Quantifying catechin at 230 nm using a UV–vis spectrophotometer, unveils information on the dynamics of controlled release that are influenced by pH and the presence of proteases. This observation indicates that the release of catechin from Cat@MHAP occurs readily upon reaching the cancer-affected tissue site (Fig. 2).^[23]^ Cat@MHAP nanoparticles demonstrated better radical scavenging (78.5%) abilities at 500 nM in DPPH tests as compared to free catechin (48.8%). Notably, MHAP and NH_2_@MHAP demonstrated no free radical scavenging.^[23]^ However, Cat@MHAP showed strong superoxide radical scavenging at 50 nM, surpassing free catechin at 500 nM. The conjugation of catechin onto MHAP nanoparticles with a large surface area increases the reactive surface, influencing chemical and biological interactions. The findings are consistent with supramolecular chemistry and nanotechnology research, demonstrating increased antioxidant reactivity following conjugation to nanoparticle surfaces. Cat@MHAP has much increased antioxidant activity, which supports the concept that assembly on MHAP nanoparticles increases biochemical reactivity Considering the encouraging antioxidant properties of Cat@MHAP, it would be helpful to investigate how well it functions in physiological settings, such as different pH and temperature ranges, in order to improve our comprehension in biological settings.

Field-emission scanning electron microscopy cross-sectional images used in the characterization of MHAP and Cat@MHAP films showed homogeneous and compact coating layers free of abrupt interfaces or fractures. Cat@MHAP (1) and Cat@MHAP (2) showed coating thicknesses of around 81.09 and 1829.72 nm, respectively. The coating thicknesses of MHAP (1) and MHAP (2) were 63.07 and 940.58 nm, respectively. AFM measurement demonstrated that Cat@MHAP had a substantially smaller root mean square surface parameter (Rq) of 4.9 nm than MHAP, which was 35.32 nm.^[24]^ This indicates that chemical modification has an effect on the surface roughness of MHAP nanoparticles.

For the use of selenium-doped hydroxyapatite against osteosarcoma, the in vivo stability of these nanomaterials should be given primary consideration. Earlier research has indicated that selenium-doped hydroxyapatite are highly stabile when tested in vivo and in vitro. These nanomaterials are biocompatible, and thus can interact with the tissues inside the body without causing side effects. The properties of these nanomaterials are enhanced by doping selenium into hydroxyapatite, which can further improve the stability in vivo. We know that selenium-doped hydroxyapatite is shaped to nanocomposites by the formation of strong bonds with bone tissue, indicating the improvement in stability, which allows integration of these materials with bone, thereby providing support and therapeutic efficacy at the same time. Nevertheless, we cannot include the possibility of lesser stability in certain cases as the stability of selenium-doped hydroxyapatite depends on various factors including selenium concentration as well as the synthesis methodologies, therefore, further studies should be conducted to confirm the stability of these nanocomposites.

## 5. Chitosan, PEG, and folate enhance drug delivery

**Figure 3.**
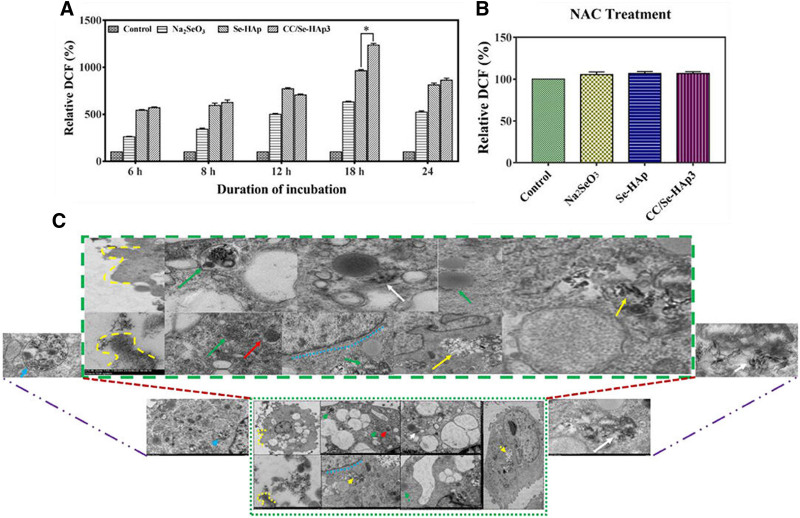
This figure depicts the generation of reactive oxygen species and their activity inside the cell after internalization. (A) ROS formation showed that catechin-modified nanomaterials (CC/Se-HAp) showed a tremendous increase in relative fluorescence intensity, which (B) was non-detectable in the presence of ROS inhibitor. (C) Cellular internalization had successfully occurred as indicated by the TEM micrographs. Reprinted from Ref. ^[15]^. doi: 10.3389/fonc.2019.00499, which is licensed under a Creative Commons Attribution 4.0 International License. ROS = reactive oxygen species.

Chitosan has been reported as unique structures which contains significant functional groups with specified chemical capabilities that make biomaterials with biodegradability and advantageous biocompatibility.^[25]^ Chitin combine to form chitosan, thus it is a polysaccharide (Fig. 3).^[26]^ This material has widely been used in artificial skin production, targeting drug as well as drug carrier. This makes the nanomaterial more effective by making them target specific and reachable to specific target by crossing the barriers. Moreover, nanoparticles coating with polyethylene glycol (PEG) improves drug efficiency as well as gene delivery to the specific target tissues and cells. PEGylating proteins have been shown to have improved decreased immunogenicity, systemic circulation time and as well as enhanced target specificity because of the PEG coatings on systemically administered nanoparticle. Similar effects have been indicated by folate such that folate coated nanomaterials are more target specific and effective as compared to those without being coated by folate time, reported that thermosensitive and in situ-formed hydrogels on the basis of PLGA–PEG–PLGA are corilagin and low-molecular-weight chitosan thus can directly be delivered to tumor cells via intratumor injection. These nanomaterials have shown higher drug infiltration and desired thermosensitive properties.

## 6. Emerging trends of nanomaterials

HAP nanoparticles, which are essential in osteosarcoma therapy, address challenges posed by conventional chemotherapeutic drugs such as low solubility, limited targeting, and multidrug resistance. Pure HAP lacks cellular recruitment and regulatory capabilities. To address these limitations, researchers have investigated composite materials that combine HAP nanomaterials with biopolymers and metal ions, enhancing physical and biological characteristics, mechanical strength, stability, and bone cell function.^[27,28]^ Several composite materials have been developed to treat localized osteosarcoma. Similarly, in vitro and in vivo investigations showed that functionalizing HAP with folic acid and loading doxorubicin improved selectivity, decreased cytotoxicity, and had substantial inhibitory effects on tumor development. Furthermore, HAP has revealed valuable in gene therapy, functioning as a nonviral gene carrier with excessive protection and effectiveness. Radioactive isotopes that operate as internal radiation sources and generate β and γ radiation, such as strontium-89 (89Sr), phosphorus-32 (32P), and gadolinium-159 (159Gd), can be added to nHAp. This dual function facilitates imaging and allows for efficient tumor removal. In addition, these isotopes can be separated from chelating agents to reduce the likelihood of toxicity by preventing buildup in healthy tissues. These radioactive nHAp materials’ combination of internal radiation therapy and imaging capabilities offers great potential for the detection and treatment of cancer.

Scientific research has shed light on the powerful effects of green tea polyphenols, notably in the fight against tumor development.^[25–28]^ These bioactive chemicals play a variety of effects, including inhibiting cell proliferation and inducing antioxidant enzymes involved in cellular defense systems. Furthermore, the anticancer capabilities of green tea catechins, particularly EGCG, have garnered a lot of consideration. Its structural features, which include numerous hydroxyl groups, contribute to its medicinal effectiveness. EGCG promotes apoptosis in cancer cells by increasing oxidative stress, inhibiting tumor necrosis factor activity, and altering key signaling pathways such as PI3K/Akt and TGF-β1/Smad. Combining EGCG with PI3K/Akt inhibitors enhances apoptosis induction, suggesting synergistic therapeutic pathways. Efforts to improve EGCG bioavailability and cellular absorption are continuing, with encapsulation inside hydrophobic nanocarriers showing encouraging results. Copper enrichment in mediums sensitizes cells to EGCG-mediated growth suppression, offering information on new techniques in cancer therapy.

The production of chitosan–iron complexes adds another degree of complexity to the investigation, with careful pH control and subsequent crosslinking with tripolyphosphate resulting in stable complexes. This rigorous approach to material production enables exact particle size management, which improves repeatability and possible scalability of nanocomposites. The EGCG enhances the contributions by exhibiting excellent complex formation and minimal structural alterations.^[28]^ The encapsulation efficiency and loading capacity tests highlight the complex relationship between material properties and production procedures. The UF method by centrifugation appears as a vital step in reducing the influence of purification on particle integrity. Considering all, the review on Se-HAp nanocomposites with catechin modifications provides fresh perspectives on targeted osteosarcoma therapy, addressing a range of topics from encapsulation to biological activity. It also plays the groundwork for future research and possible clinical trials.

Conventional treatment options being used for osteosarcoma include chemotherapy, radiation therapy, and surgery. These methods are considered selectively effective, however, there are several drawbacks associated with these treatment options being used for osteosarcoma. Such that surgery is invasive, thereby inducing damage to healthy tissues and even organs. Similarly, radiation therapy and chemotherapy pose severe side risks to functioning organs and have severe side effects on the body. Therefore, nanomaterials based therapeutic options are considered a suitable replacement for treatment strategies being used against osteosarcoma. Selenium-doped hydroxyapatite in this regard offers tremendous advantages when compared with traditional treatment options. For instance, these nanocomposites are biocompatible, and thus integrate into the tissues, implicating a minimal rejection risk. In addition, doping of selenium induces antitumor properties of hydroxyapatite, thereby making the nanocomposite even more effective against osteosarcoma by inhibiting the growth of cancer cells. Selenium-doped hydroxyapatite depicts a higher therapeutic potential, therefore, can be considered as the suitable treatment option for osteosarcoma as compared to several conventional therapeutics. These nanomaterials are target specific with qualities of being biocompatible and less invasive, thereby posing fewer side effects. Overall, these characteristics of selenium-doped hydroxyapatite nanocomposites make them efficient and effective, thus can be used as promising therapeutic options for clinical applications as well as research studies.

## 7. Conclusion and future perspectives

Firstly, the development of nanoparticles with sizes around 200 nm based on chitosan–iron complexes, which incorporate chitosan derivatives with polyethylene glycol and folic acid, opens the door to encapsulating hydrophilic active ingredients such as EGCG with high encapsulation efficiency and loading capacity. However, greater investigation into release mechanisms, kinetics, and the fate of released particles under different circumstances is required for clinical translation. Secondly, there has to be improvement in targeted ligand selection and techniques in nano-drug delivery systems for osteosarcoma therapy. This includes figuring out which biomarkers are specific to bone tumor cells and creating smart ligands that maximize cytotoxic potential while reducing off-target effects. Targeting effectiveness and treatment efficacy may be improved by investigating intracellular reactions to ligand–receptor binding and modifying multiple ligands. Finally, there is a need to look at nano-drug delivery approaches for circulating tumor cells for both therapeutic and diagnostic applications. Patients with osteosarcoma may have better overall treatment results and a significant reduction in tumor metastasis by the targeted collection and treatment of circulating tumor cells. With enhancement and development of nanotherapeutics approaches by using nanotechnology, and novel nanoparticles the treatment of osteosarcoma can be made promising and effective. These novel nanoparticles have shown high potential by presenting excellent properties to improve the therapeutic efficacy for osteosarcoma and osteogenesis side by side. There is a need that nanoparticles should be combined with various functional molecules, peptides and other proteins as well as drugs to achieve improved and promising therapeutic effects against osteosarcoma.

Researchers and scientists should further investigate the enhanced efficacy by finding the optimal concentration of selenium content in the selenium-doped hydroxyapatite nanocomposites. These investigations will further help in maximizing the stability as well as antitumor efficacy of these nanocomposites in vivo. A wide range of studies should also be conducted for the improvement and enhancement of synthesis and designing of these nanocomposites, which can further enhance the delivery and efficacy of these nanomaterials against osteosarcoma. More importantly, the materials can be combined with additional antitumor molecules such as catechins, which have shown effectiveness in different cancer types. Last but not the least, materials scientists should cooperate with oncologists for the development of more refined nanocomposites that help the researchers to determine effective and promising treatment options by combining the properties of selenium-doped hydroxyapatite with other therapeutic molecules.

## Author contributions

**Conceptualization:** Tao Degang, Xu Wei, Li Haitang, Zhou Rong.

**Data curation:** Tao Degang, Xu Wei.

**Formal analysis:** Xu Wei, Jiang Chenyu.

**Investigation:** Lai Aining, Jiang Chenyu.

**Methodology:** Lai Aining.

**Resources:** Lai Aining.

**Software:** Lai Aining.

**Writing – original draft:** Tao Degang, Xu Wei, Shen Jianying, Lai Aining, Jiang Chenyu, Zhou Rong.

**Writing – review & editing:** Shen Jianying, Li Haitang, Zhou Rong.
